# Characterizing rhizosphere microbial communities associated with tolerance to aboveground herbivory in wild and domesticated tomatoes

**DOI:** 10.3389/fmicb.2022.981987

**Published:** 2022-09-14

**Authors:** Emily Tronson, Ian Kaplan, Laramy Enders

**Affiliations:** Department of Entomology, Purdue University, West Lafayette, IN, United States

**Keywords:** rhizosphere, microbiome, tolerance, herbivory, tomato

## Abstract

Root-associated microbial communities are well known for their ability to prime and augment plant defenses that reduce herbivore survival or alter behavior (i.e., resistance). In contrast, the role root microbes play in plant tolerance to herbivory, an evolutionarily sustainable alternative to resistance, is overlooked. In this study, we aimed to expand our limited understanding of what role rhizosphere microbial communities play in supporting tolerance to insect damage. Using domesticated tomatoes and their wild ancestors (*Solanum* spp.), we first documented how tobacco hornworm (*Manduca sexta*) herbivory impacted tomato fruit production in order to quantify plant tolerance. We then characterized the bacterial and fungal rhizosphere communities harbored by high and low tolerance plants. Wild tomatoes excelled at tolerating hornworm herbivory, experiencing no significant yield loss despite 50% leaf area removal. Their domesticated counterparts, on the other hand, suffered 26% yield losses under hornworm herbivory, indicating low tolerance. Ontogeny (i.e., mid- vs. late-season sampling) explained the most variation in rhizosphere community structure, with tomato line, tolerance, and domestication status also shaping rhizosphere communities. Fungal and bacterial community traits that associated with the high tolerance line include (1) high species richness, (2) relatively stable community composition under herbivory, and (3) the relative abundance of taxa belonging to *Stenotrophomonas*, *Sphingobacterium*, and *Sphingomonas*. Characterizing tolerance-associating microbiomes may open new avenues through which plant defenses are amended in pest management, such as plant breeding efforts that enhance crop recruitment of beneficial microbiomes.

## Introduction

In response to herbivory, plants rely on a blend of defensive mechanisms to limit herbivore feeding and mitigate fitness losses. The former is achieved through resistance traits, which reduce herbivore fitness (antibiosis) or affect herbivore behavior such that the herbivore less successfully colonizes a plant (antixenosis) (Painter, 1951; Kogan and Ortman, 1978). Resistance mechanisms effectively combat herbivore damage, but can also lead to cycles of escalating defenses (Dawkins and Krebs, 1979) and resistant insect populations (Gould, 1998). Resistance may incite defensive arms races, but its counterpart, tolerance, is thought to place little, if any, selection pressure on herbivores. Instead, tolerance mechanisms involve plant processes that minimize fitness losses without targeting herbivore biology or behavior (Stowe et al., 2000; Rausher, 2001; Espinosa and Fornoni, 2006; but see Poveda et al., 2012). Tolerance traits can involve, for example, moving resources away from sites of herbivore feeding, increasing photosynthetic efficiency to fuel compensatory growth, or mitigating oxidative stress during herbivory (Strauss and Agrawal, 1999; Stowe et al., 2000; Koch et al., 2016). Despite being an evolutionarily stable mediator of plant-herbivore interactions and an integral component of integrated pest management (Pedigo and Higley, 1992; Peterson et al., 2018), tolerance to herbivory remains an understudied realm of plant defenses (Peterson et al., 2017).

Expressing tolerance involves responses in both shoots and roots. Roots are particularly important because they serve as sites of nutrient storage and mobilization that buffer against the fitness consequences of above-ground herbivore damage (Orians et al., 2011). Roots play another critical role, however, that is rarely discussed alongside plant tolerance; roots actively release photoassimilates, mucilage, secondary metabolites, and other exudates into the rhizosphere (Bais et al., 2006; Loyola-Vargas et al., 2007), creating a nutrient-rich zone that attracts soil microbes (Schenck zu Schweinsberg-Mickan et al., 2012). By tailoring root exudation to attract certain microbes, plants may be able to enrich this rhizosphere community with beneficial microbial partners that increase plant resilience to biotic stresses such as herbivory (Bulgarelli et al., 2013; Pieterse et al., 2014). Currently, we have a foundational awareness of how rhizosphere communities contribute to resistance to herbivory, notably by priming plant defenses (Pieterse et al., 2014), augmenting secondary metabolite production (Korenblum and Aharoni, 2019; Rivero et al., 2021), and influencing plant volatile profiles to attract natural enemies (Guerrieri et al., 2004; Fontana et al., 2009; Hempel et al., 2009). However, our understanding of how this community affects plant tolerance to herbivory is lacking (Vannette and Hunter, 2009).

Root microbial communities have the potential to play powerful roles in tolerance responses. In plants exposed to herbivory, single root microbes or small consortia can contribute to maintaining shoot and root biomass (Cosme et al., 2016; Bernaola and Stout, 2021; Contreras-Cornejo et al., 2021) and yields (Herman et al., 2008; Bernaola and Stout, 2021), improve resource reallocation (Frew et al., 2020), increase leaf chlorophyll content (He et al., 2021), and reduce oxidative stress (Selvaraj et al., 2020). These studies clarify how root microbes may alter host expression of tolerance and identify promising candidates for tolerance-promoting microbial mutualisms. However, to our knowledge, previous experiments exclusively consider a rhizosphere consisting of between one and six taxa, rather than the hundreds of taxa that naturally make up a root microbiome (French et al., 2021a). The absence of research on what role whole root microbiomes play in plant tolerance neglects microbial interactions and emergent functions at the whole-community level. Studies considering such root microbiomes are needed to understand how microbial communities can be leveraged to buoy plants through biotic stresses in field environments.

Identifying how root microbiomes mediate plant tolerance to insect damage provides new opportunities to improve crop protection and pest management in agroecosystems. Through the process of selection for desirable phenotypes, crop domestication has unintentionally altered plant communication with a root microbial community (Olsen and Wendel, 2013; Pérez-Jaramillo et al., 2016; Jaiswal et al., 2020; Soldan et al., 2021). In many cases, domestication has reshaped root architecture and exudation patterns (Iannucci et al., 2017) and diminished crop ability to benefit from growth promoting microbes (Jaiswal et al., 2020). This loss of plant-microbe functionality may help explain the increased susceptibility of domesticated cultivars to insect herbivores (Chen et al., 2015; Whitehead et al., 2017). If wild plants evolved to foster beneficial microbiomes that enhance their tolerance of herbivory, shifts in root traits and exudation patterns brought about by domestication could bring consequences for crop tolerance of insect damage.

In this study, we investigated the relationship between fungal and bacterial rhizosphere community characteristics and tolerance to herbivory using four wild and domesticated tomato lines (*Solanum* spp.). We chose to work within a tomato domestication spectrum because previous research has indicated that domesticated tomatoes are less capable of establishing microbial mutualisms and also possess lower tolerance to herbivory than their wild relatives (Welter and Steggall, 1993; Paudel et al., 2019; Ferrero et al., 2020; Jaiswal et al., 2020). In a field environment, we first screened tomato lines for tolerance to herbivory using the specialist tobacco hornworm (*Manduca sexta*), which triggers strong tolerance responses in tomatoes (Steinbrenner et al., 2011). We then characterized the fungal and bacterial root microbial communities associating with lines expressing high and low tolerance using a standard metabarcoding approach. Overall, we predicted that rhizospheres of wild and/or high tolerance lines would be more diverse and exhibit stronger shifts in community composition in response to herbivory compared to rhizospheres of domesticated and/or low tolerance cultivars.

## Materials and methods

### Experimental design

Modern tomato cultivars (*Solanum lycopersicum*) are the products of initial domestication efforts in South America, likely involving the wild *Solanum pimpinellifolium* (Ranc et al., 2008; Blanca et al., 2015). From this wild species, selection for larger fruit size yielded the domestication intermediate, *Solanum lycopersicum* var. *cerasiforme*, or ‘Matt’s Wild Cherry,’ though some modern genotypes are the likely product of hybridization between *S. pimpinellifolium* and *S. lycopersicum* (Ranc et al., 2008; Blanca et al., 2015, 2022). As is the case in most domestication events (Chen et al., 2015), artificial selection for commercially desirable traits in cultivated tomatoes has reduced genetic diversity (Ranc et al., 2008) and inadvertently dampened resistance (Bai and Lindhout, 2007; Li et al., 2018; Ferrero et al., 2020) and tolerance (Welter and Steggall, 1993; Ferrero et al., 2020) to herbivory.

To represent this spectrum of domestication, as well as an anticipated spectrum of tolerance, we used two domesticated (*S. lycopersicum* ‘Better Boy’ and ‘Sioux’) and two wild tomato lines (fully wild *S. pimpinellifolium* accession WVa700 and domestication intermediate *S. lycopersicum* var. *cerasiforme*). All four lines are indeterminate, red-fruited tomatoes. Both domesticated cultivars are slicing tomatoes and both wild lines morphologically resemble cherry tomatoes. Domesticated cultivar and intermediate ‘Matt’s Wild Cherry’ seeds were purchased from Johnny’s Selected Seeds (Winslow, ME) in 2020, while the remaining *S. pimpinellifolium* seeds were sourced from the Iyer-Pascuzzi Lab at Purdue University. Originally, three additional wild tomato species, *Solanum peruvianum*, *Solanum chilense*, and *Solanum chmielewskii*, were included in this field experiment. However, these three species began fruiting only 2 weeks before the season’s first frost. Because of the lack of fruit data, and resultant inability to estimate tolerance, these three lines were removed from analyses.

Seeds were sown in seedling flats and kept in a greenhouse at the Throckmorton Purdue Agricultural Center (TPAC) in Lafayette, IN during the summer of 2020. After 5 weeks, at which point seedlings had three to five leaves, we moved plants to a shadehouse for 1 week of hardening off. Seedlings were then hand transplanted to a field at the Meigs Horticultural Farm at TPAC with drip irrigation and fertilizer (1000# 9-23-30 and 156# 46-0-0) applied 2 months prior. The field was organized into ten rows, each containing five blocks of eight plants belonging to one of each tomato line (*n* = 4) by infestation (*n* = 2) treatment and organized in a randomized complete block design. Rows were spaced six feet apart with plants within rows spaced five feet apart. Dead seedlings were replaced as necessary in the week following transplant. Metal stakes were then added along the row center at an interval of every two plants and used to trellis plants with nylon twine using the Florida Weave method.

### Tobacco hornworm infestation

We began the hornworm infestation treatment 5 weeks after transplant, during flowering but before fruiting. Hornworms were reared from eggs sourced by a commercial supplier (Great Lakes Hornworms; Romeo, MI) at room temperature on artificial diet. Once they reached second to third instars, hornworms were moved to the field and placed in nylon nets that we secured to tomato leaves with twist ties to avoid caterpillar movement onto neighboring plants. We aimed to defoliate 50% of leaf area across a spectrum of leaf age. We selected this defoliation threshold to exceed the point at which Welter and Steggall (1993) saw yield suffer as a result of manual defoliation in tomatoes (30% defoliation). Initially, nets were secured to the second tomato true leaf. Once hornworms had eaten the netted leaf, the net was moved to a new, higher leaf on the opposite side of the plant. Plants typically held two nets containing two hornworms each at a time, with smaller plants receiving fewer nets, and larger plants receiving more. Once a plant reached 50% defoliation (visually estimated), we removed all hornworms from the plant. After 3 weeks of herbivory treatments, if a plant had still not reached this defoliation threshold, hornworms were removed and the plant was hand-defoliated by stripping leaflets from the rachis in order to reach 50% defoliation. Any wild hornworms that colonized plots were either placed in nets and relocated to large, infested treatment plants when herbivory treatments were ongoing, or removed from the field. Background damage level in the uninfested control plants averaged < 10% and no other herbivores were routinely observed on plants that could confound the treatment effect.

### Rhizosphere sampling

The 50 blocks in this field experiment were randomly assigned to one of three sampling groups, separated by the seasonal timepoint at which destructive rhizosphere samples were taken for bacterial and fungal community profiling. We designed block size to account for environmental variation in the field. Due to spatial and logistical limitations associated with destructive rhizosphere sampling, blocks did not include timepoint (i.e., separate blocks were allocated for each seasonal timepoint). The first group (*n* = 15 blocks, hereafter “early season”) was sampled for rhizosphere soil immediately after the conclusion of the hornworm herbivory treatments (28 July 2020) in order to capture immediate changes that herbivory induced in tomato rhizospheres. Because herbivory treatments concluded before plants began fruiting, plants belonging to this early season group have rhizosphere data, but no fruit data. The second group (*n* = 25 blocks, hereafter “late season” or “truncated lifespan”) was also sampled for rhizosphere soil, but this sampling point took place later in the season (2 September, 25 September, and 2 October 2020). In order to standardize rhizosphere samples across line ontogeny, we sampled lines within these blocks 4 weeks after 80% of plants of a given line had produced at least one mature fruit. By re-sampling rhizosphere communities at this second time point, we aimed to isolate the life-long impacts of hornworm herbivory on tomato rhizosphere communities. This late season or truncated lifespan group has both rhizosphere data and fruit data (up until the point of rhizosphere harvests). The third group (*n* = 10 blocks, hereafter “full lifespan”) was never sampled for rhizosphere soil, but instead allowed to fruit until senescence (i.e., first frost). By keeping these ten blocks until the season’s end, we accounted for herbivory-induced shifts in seasonal fruiting patterns. Some have observed infested plants responding to herbivory by prolonging senescence (i.e., extending a fruiting window) in both non-solanaceous (Weinig et al., 2003; Martin et al., 2015) and solanaceous plants (Welter and Steggall, 1993; Schwachtje et al., 2006). In including this final, full lifespan group, we ensured that we were not underestimating plant yield, a proxy for fitness, in the hornworm infested treatment. Because rhizosphere samples were never taken, we have comprehensive fruit data, but not rhizosphere data, for this full lifespan group.

To collect rhizosphere soil, we first uprooted selected plants and removed any large soil clumps from the roots. We then cut primary and lateral roots with ethanol-sterilized scissors and placed roots in a plastic bag. These bagged roots were hand-shaken and massaged to dislodge as much root-surface soil as possible, after which roots were discarded. From the remaining soil, three samples were collected in sterile 1.5 ml Eppendorf tubes and temporarily stored in a cooler. Upon returning from the field, these samples were transferred to a –80°C freezer, where they remained until DNA extraction.

### Bulk soil analysis

To assess if soil characteristics varied across the field, we collected about 1 cup of bulk soil from each uprooted plant on the day of that plant’s rhizosphere sampling. Soil from plants of the same block were then pooled and subsampled. Because the 10 full lifetime blocks were never sampled for rhizosphere soil, we never collected bulk soil from these blocks. The resultant 40 blocked samples were sent to A&L Great Lakes Laboratories, Inc. (Fort Wayne, IN) for analysis of the following: organic matter, P, K, Mg, Ca, soil pH, and cation exchange capacity (CEC).

### Fruit harvest

We harvested mature (i.e., red) fruits from all tomato plants on a weekly basis. For each plant, we recorded two measurements: (1) yield (i.e., a count of fruit removed) and (2) weight (i.e., the total weight of all harvested fruit). In the case of especially prolific plants (i.e., late-season ‘Matt’s Wild Cherry’ and *S. pimpinellifolium*), we estimated yield; specifically, after harvesting and weighing fruits, we separated 150 fruits and weighed this subset to estimate total fruit count, assuming that average fruit weight from this subset was representative of the total.

### DNA extraction and sequencing

DNA from 0.25 g of rhizosphere soil was extracted with Qiagen DNeasy PowerSoil HTP 96 kits, according to manufacturer instructions. Samples were sent to the University of Minnesota Genomics Center for library preparation and sequencing of the V4 and ITS2 region of the 16S rRNA and ITS, respectively, according to the methods outlined in Gohl et al. (2016). Sequencing was performed on the Illumina MiSeq with V3 chemistry and paired end 300 bp sequencing. Primers targeting the V4 region were 515F GTGCCAGCMGCCGCGGTAA and 806R GGACTACHVGGGTWTCTAAT (Caporaso et al., 2011), and primers targeting the ITS2 region were 5.8SR TCGATGAAGAACGCAGCG and ITS4 TCCTCCGCTTATTGATATGC (White et al., 1990). Sample demultiplexing was performed by the University of Minnesota Genomics Center with Illumina software. Total microbial biomass was estimated using PicoGreen dsDNA quantification by the University of Minnesota Genomics Center.

### Sequence pre-processing

16S rRNA sequencing of the V4 region produced 13.63 million reads, while ITS sequencing of the ITS2 region produced 11.65 million reads. Adapter removal and primer clipping was performed with Trimmomatic (v. 0.36) (Bolger et al., 2014) and Cutadapt (v. 1.13) (Martin, 2011). Reads were subsequently processed through the dada2 (v 1.14.1) (Callahan et al., 2016) pipeline by filtering and trimming reads based on quality for 16S rRNA sequences, estimating error rates, merging paired end reads, and removing chimeras. Taxonomy was assigned with the Silva reference database (v. 138.1) (Quast et al., 2012) for 16S sequences and UNITE (v. 8.3) (Nilsson et al., 2019) for ITS sequences. Likely contaminant sequences, archaeal, mitochondrial, and plastid 16S sequences, as well as low abundance sequences (fewer than 2 reads across 5% of samples) were filtered out, leaving 2,173 bacterial amplicon sequence variants (ASVs) and 452 fungal ASVs (Davis et al., 2018). After preprocessing and removing samples with fewer than 2,000 16S reads, the dataset contained 326 samples. For our ITS dataset, we removed samples with fewer than 1,000 reads, leaving 2.49 million reads in the dataset across 329 samples, averaging 16,495 reads per sample (Supplementary Table 1). Pre-processing followed methods developed by French et al. (2021a).

### Statistical analysis

#### Quantifying tolerance to herbivory

Tolerance to herbivory has been functionally defined as a plant’s fitness loss per unit damage (Stowe et al., 2000). This means that, on the simplest level, two values are required to estimate tolerance: herbivore damage and plant fitness. While quantifying herbivore damage can be straightforward when working with leaf-eaters such as the tobacco hornworm, total plant fitness is challenging and laborious to capture. Therefore, most methods of quantifying tolerance establish some proxy for plant fitness (Strauss and Agrawal, 1999). For our purposes, we used two proxies: fruit yield (i.e., total number of tomatoes produced) and single fruit weight. Both of these metrics are common proxies for tolerance (count: Weinig et al., 2003; Martin et al., 2015; Lin et al., 2021; weight: Welter and Steggall, 1993; Olejniczak, 2011). Though frequently used, fruit count and weight are just two of many plant traits that shape fitness. Other components of fitness, such as seed count or more subtle traits like attractiveness to pollinators (Stowe et al., 2000) may also contribute to plant tolerance. Therefore, fruit count and/or weight serve only as possible and partial estimates of plant fitness.

To estimate tolerance, the fruit count or weight of infested plants was first divided by the fruit count or weight of uninfested plants. The log of this quotient produces a tolerance log-effect size:


$$t\,o\,l\,e\,r\,a\,n\,c\,e=\text{log}\,\left(\frac{i\,n\,f\,e\,s\,t\,e\,d\,\left[y\,i\,e\,l\,d\,O\,R\,f\,r\,u\,i\,t\,w\,e\,i\,g\,h\,t\right]}{u\,n\,i\,n\,f\,e\,s\,t\,e\,d\,\left[y\,i\,e\,l\,d\,O\,R\,f\,r\,u\,i\,t\,w\,e\,i\,g\,h\,t\right]}\right)$$

In this formula, infested and uninfested values describe either a single sampling point (i.e., a given plant in a given week and block) at the smallest level or seasonal summaries (e.g., a sum of lifetime yield for all plants in a line) at the largest. Under this quantification of tolerance, a value of zero indicates perfect compensation, in which infested and uninfested plants produce the same number or weight of fruit, on average, in a given time frame. Negative values reflect low-tolerance, whereby infested plants produce fewer or lighter fruit than uninfested plants, reflecting a fitness cost to herbivory incurred by the plant. Positive values indicate overcompensation, in which infestation increases fruit number or weight with respect to undamaged plants; in this case, herbivory treatments increase plant fitness.

When performing weekly fruit harvests, one or both infestation treatment plants of a given tomato line and block sometimes matured no fruit in the week. In these cases, estimating tolerance at the sampling-point-level (i.e., line * block * week), produces undefined numbers, as zero is in the numerator and/or denominator. To avoid undefined tolerance estimates when plotting and analyzing weekly fruiting trends, we added a constant of one to all fruit count entries. This addition affects figures and statistical models that examine weekly fruiting trends, but not those that handle lifetime fruit metrics, as zeros did not exist in lifetime fruit summaries. During the field season, time constraints and high yields led to infrequent sampling of *S. pimpinellifolium* plants in eight blocks. Consequently, these plants were removed from figures and statistical analyses. In addition, two outliers at the sampling point level were removed where recorded yields were more than six standard deviations away from the mean and likely recording errors.

To characterize the tolerance of our four tomato lines, we examined the impact of herbivory on lifetime summaries of our two fitness proxies, yield and single fruit weight, by fitting linear mixed models to each proxy. Both variables were normally distributed. Because both fruit weight and lifetime yield vary considerably between our four tomato lines, we fit separate models for each line. We also fit separate models for truncated (*n* = 25 blocks) and full lifespan (*n* = 10 blocks) sampling timepoints (i.e., 4 lines × 2 proxies × 2 timepoints = 16 models). Within these models, herbivory (infested or uninfested) was treated as a fixed effect and block was treated as a random effect. We used these statistical analyses as well as comparisons of yield effect size between herbivory treatments in order to categorize our four tomato lines into three tolerance categories (high, intermediate, or low). We also considered whether expression of tolerance varied across the season for any of our lines. To do so, we fit a linear mixed model to sampling-point-level tolerance estimates (i.e., line * block * week) with tomato line and week treated as fixed effects and block treated as a random effect. For this model, we used only the tolerance estimates from the full lifespan group (*n* = 10 blocks). All analyses were performed in the R statistical environment (v. 4.1.0) (R Core Team, 2021). Models were built using the nlme package (v. 3.1–153) (Pinheiro et al., 2013) function lme(), and diagnostics were created using both nlme and lme4 (v. 1.1–27.1) (Bates et al., 2015).

To assess a potential nutritional gradient that may explain spatial variation in tolerance across our field, we used ggplot2 (v. 3.3.5) (Wickham, 2016) to create heatmaps of block-level summaries of organic matter, P, K, Mg, Ca, soil pH, and CEC obtained from bulk soil samples. We also conducted canonical correspondence analyses (CCAs) to evaluate the extent to which block-level soil nutritional factors (organic matter, P, K, Mg, Ca, soil pH, and CEC; constraining variables) explained variation in bacterial and fungal Bray–Curtis dissimilarity (response variable). We used vegan (v. 2.6–2) (Oksanen et al., 2022) to conduct the two CCAs and assessed the significance of CCA terms with vegan’s anova.cca(). All figures were made using the ggplot2 package and Inkscape (v. 1.1.2). Fruit count and weight data, as well as code for statistical analysis and figure generation, can be found at https://github.itap.purdue.edu/LaramyEndersGroup/Tomato-Tolerant-Rhizosphere-Microbiomes.

#### Rhizosphere community analyses

To examine changes in tomato rhizosphere communities across treatments, we first calculated standard microbial community diversity metrics, including inverse Simpson diversity, richness, Shannon diversity, and Bray–Curtis dissimilarity, using the phyloseq package (v. 1.38.0) (McMurdie and Holmes, 2013). We then compared these alpha- and beta-diversity metrics from bacterial (16S) and fungal (ITS) communities across tomato lines, herbivory treatments, and seasonal timepoints. To do so, we utilized two models that categorized our four tomato lines on either their domestication status (wild or domesticated) or tolerance to herbivory (high, intermediate, or low). These models included herbivory (infested or uninfested), seasonal timepoint (early or late), and line (‘Sioux,’ ‘Better Boy,’ ‘Matt’s Wild Cherry,’ and *S. pimpinellifolium*) nested within either tolerance category or domestication status.

To compare rhizosphere community structure, we visualized differences in beta diversity with Principal Coordinates Analysis (PCoA). PCoA ordination was created using Bray–Curtis dissimilarity and the ordinate() function in the phyloseq package. We then conducted a PERMANOVA of Bray–Curtis distance with the adonis2() function in vegan. Here, infestation, seasonal timepoint, and line nested within either tolerance or domestication status were used as fixed factors after reads were scaled to the smallest library size. Where significant interactions occurred, we then developed separate models, for example by separating tomato lines within both bacterial and fungal datasets.

To compare microbial species richness and evenness (i.e., Shannon diversity, observed species richness, inverse Simpson diversity), we first determined if assumptions of normality and homogeneity of variance could be met. When assumptions were met, we conducted an ANOVA of linear models fit to alpha-diversity metrics with herbivory, seasonal timepoint, and line nested within either domestication or tolerance category included as fixed factors. When assumptions could not be met, we performed a non-parametric, rank-based ANOVA using the raov() function in Rfit (0.24.2) (Kloke and McKean, 2012). Because the raov() function cannot incorporate nesting terms, we ran separate models that included herbivory, seasonal time point, and either line, tolerance category, or domestication status.

DESeq2 (v. 1.34.0) (Love et al., 2014) was used to identify differentially abundant bacterial and fungal ASVs and families between tolerance categories (high, intermediate, or low), domestication status (wild or domesticated), and herbivory treatments (infested or uninfested) within either tolerance category or domestication status. Within each analysis, the Benjamini and Hochberg (1995) false discovery rate was implemented to correct for multiple comparisons. Finally, to identify the impact of our treatments on overall rhizosphere microbial biomass, we estimated total microbial dsDNA concentration of each soil sample using PicoGreen. These dsDNA ng/μl concentrations were standardized to the weight of soil samples used for extractions (0.19313–0.28238 g) to obtain a measurement of ng dsDNA/g soil for each sample. We then fit a linear model to this estimate of microbial biomass that included time point of rhizosphere sampling, herbivory, and line nested within either domestication or tolerance serving as fixed effects.

Code for rhizosphere community analysis and figure generation can be found at https://github.itap.purdue.edu/LaramyEndersGroup/Tomato-Tolerant-Rhizosphere-Microbiomes. All sequence data generated from this study have been deposited into the National Center for Biotechnology Information Sequence Read Archive under the BioProject number PRJNA849200.

## Results

### Wild tomato lines are more tolerant to herbivory than their domesticated descendants

**FIGURE 1:**
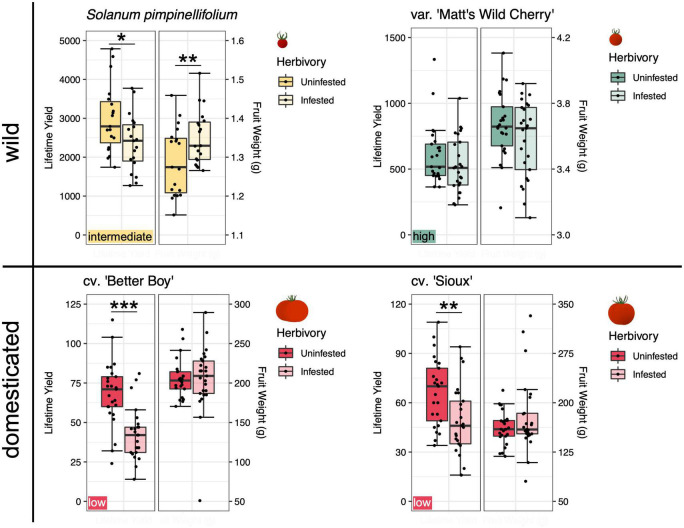
Lifetime yield and single fruit weight of individual plants from wild and domesticated tomato lines, separated by herbivory treatment (tobacco hornworm infested or uninfested). Yield and fruit weight from truncated, but not full, lifespan groups are shown. The left-hand *y*-axis depicts fruit yield, while the right-hand *y*-axis depicts fruit weight. Note the changing y-axes across panels due to variation in yield and fruit weight across tomato lines. Each line is represented by *n* = 25 blocks, except for *S. pimpinellifolium*, which is represented by *n* = 19 blocks. Significant differences in yield between herbivory treatments are indicated (see Supplementary Table 2): * represents *p* < 0.05, ** represents *p* < 0.01, and *** represents *p* < 0.001. Tolerance categories for each line are included in panel corners.

Herbivory reduced the lifetime yield of all four tomato lines to varying extents (Figure 1). Significant effects of herbivory were found only in truncated lifespan groups, and never in full lifespan groups (Supplementary Table 2). As anticipated, the two domesticated cultivars ‘Sioux’ and ‘Better Boy’ both experienced a highly significant yield reduction in truncated lifespan groups when defoliated by tobacco hornworms [Sioux: *F*_(1,__33)_ = 9.240, *p* = 0.006; Better Boy: *F*_(1,__33)_ = 36.664, *p* < 0.001; Supplementary Table 2). The average lifetime yield from cultivars under the hornworm herbivory treatment was 26% lower than that of uninfested cultivars across seasonal timepoints (i.e., truncated and full lifespan). Following this domesticated cohort in yield losses to herbivory was the wild *S. pimpinellifolium*, which yielded 14% fewer fruit across timepoints when infested, producing a significant effect of herbivory in truncated lifespan groups [*F*_(1,__25)_ = 7.102, *p* = 0.016; Supplementary Table 2]. In contrast, the domestication intermediate, ‘Matt’s Wild Cherry,’ saw negligible (3%), non-significant yield losses under herbivory [truncated lifespan: *F*_(1,__33)_ = 2.745, *p* = 0.111; Supplementary Table 2].

While hornworm infestation strongly shaped the lifetime yield of several tomato lines, fruit weight was similarly affected only for the wild *S. pimpinellifolium* (Figure 1). Specifically, infestation increased *S. pimpinellifolium* fruit weight by 5% in truncated lifespan groups [*F*_(1,__25)_ = 13.434, *p* = 0.002; Supplementary Table 2]. In the remaining three tomato lines, fruit weight did not differ significantly between infested and uninfested plants.

**FIGURE 2:**
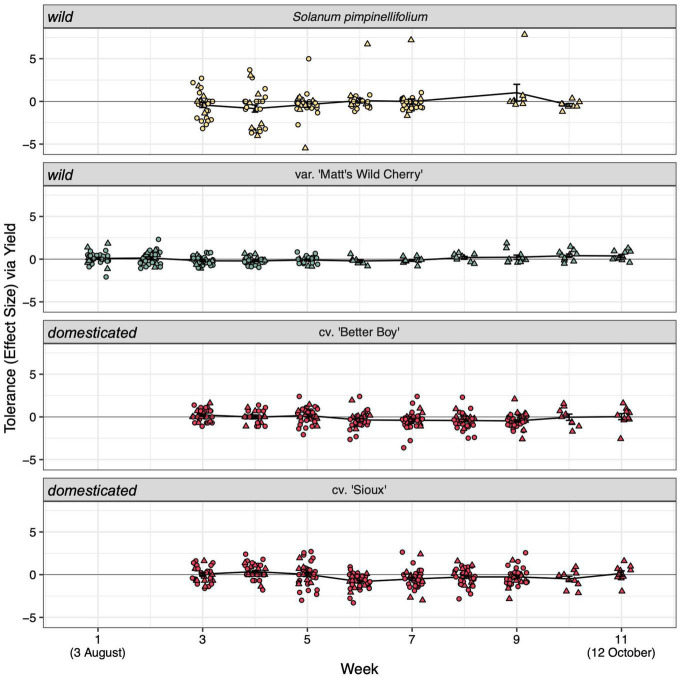
Weekly estimates of tolerance to hornworm herbivory among our four tomato lines. Domestication status (wild or domesticated) is noted on the left-hand side of panels. See Supplementary Table 3 for full statistical analyses. Tolerance scores compare infested and uninfested yields within a given block and week (see formula in section “Materials and methods”). Values of 0 reflect perfect tolerance, negative values reflect low tolerance, and positive values reflect overcompensation. Tolerance estimates were calculated with the addition of a constant (= 1) to avoid undefined scores (see section “Materials and methods” for details). Point color reflects the line’s tolerance category (Figure 1). Circles represent blocks that were destructively sampled before the season’s end for rhizosphere sampling (truncated lifespan) while triangles represent the *n* = 10 blocks allowed to naturally senesce (full lifespan).

We also found little evidence that hornworm herbivory shifted seasonal fruiting windows in any of the four considered tomato lines. Specifically, we found no effect of week on tolerance estimates within the *n* = 10 full lifespan blocks [*F*_(1,__315)_ = 0.484, *p* = 0.487; Figure 2, Supplementary Figure 1, and Supplementary Table 3]. No obvious gradient in soil organic matter, P, K, Mg, Ca, soil pH, and/or CEC that may explain spatial variation in fruiting patterns was apparent in our heatmaps (Supplementary Figure 2). However, several soil nutritional factors significantly shaped bacterial (organic matter, potassium, calcium, soil pH, and CEC; *p* < 0.01) and fungal community composition (organic matter, potassium, soil pH, and CEC; *p* < 0.05) (Supplementary Figure 3 and Supplementary Table 4). While statistically significant, these constraining variables captured only 3.77 and 3.58% of variation in bacterial and fungal communities, respectively (Supplementary Table 5).

To categorize the tolerance of each tomato line, we considered each line’s ability to maintain yields under herbivory commensurate with those of uninfested plants (i.e., yield effect size in response to herbivory) as well as the statistical significance of the herbivory treatment’s effect on yield. We categorized the domesticated cultivars ‘Sioux’ and ‘Better Boy’ as low tolerance lines, since both of these cultivars experienced significant (*p* < 0.006; Supplementary Table 2) yield losses in truncated lifespan groups and substantial (26%) yield losses overall to hornworm herbivory. Having sustained negligible (3%) and non-significant (*p* = 0.111; Supplementary Table 2) yield losses, ‘Matt’s Wild Cherry’ was categorized as a high tolerance line. Finally, although *S. pimpinellifolium* experienced significant (*p* = 0.016; Supplementary Table 2) yield losses in truncated lifespan groups and sizeable, 14% yield losses overall, the wild species also compensated with a subtle (5%) but significant increase in fruit weight in truncated lifespan groups (*p* = 0.002; Supplementary Table 2). Therefore, we categorized this line as having intermediate tolerance. These three categories were used for later rhizosphere analyses. They are helpful in allowing us to identify correlations between expressed tolerance and rhizosphere characteristics, but limited in that these three categories are populated by at most two lines each.

### Rhizosphere responses to tomato line, herbivory, and plant ontogeny

#### Tomato lines harbor unique rhizospheres that change over time

**FIGURE 3:**
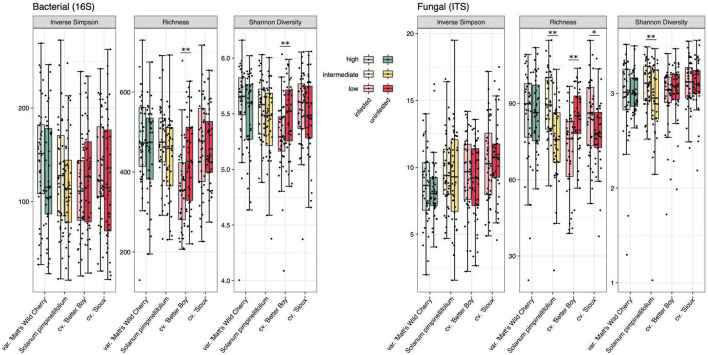
Bacterial (left) and fungal (right) alpha diversity (as represented by inverse Simpson, richness and Shannon diversity) of four tomato lines separated by tobacco hornworm herbivory treatments. *n* = 38–40 for all treatments. Significant differences in alpha diversity between herbivory treatments are indicated (see Supplementary Table 7): * denotes *p* < 0.05 and ^**^ denotes *p* < 0.01.

As expected, tomato line was a significant determinant of rhizosphere fungal and bacterial community composition in both tolerance and domestication models [tolerance model: ITS: *F*_(3,__314_ = 4.406, *p* = 0.001; 16S: *F*_(3,__317)_ = 2.742, *p* = 0.001; Supplementary Figure 4 and Supplementary Table 6]. Line had similar influence over bacterial community richness and evenness; specifically, we observed a significant effect of line on bacterial Shannon diversity [tolerance model: *F*_(3,__317)_ = 4.250, *p* = 0.006] and richness [tolerance model: *F*_(3,__317)_ = 18.726, *p* < 0.001], but not inverse Simpson diversity [tolerance model: *F*_(3,__317)_ = 1.003, *p* = 0.392] in both models. Within fungal communities, line significantly shaped all three alpha-diversity metrics [Shannon: *F*_(3,__314)_ = 3.261, *p* = 0.022; Richness: *F*_(3,__314)_ = 5.295, *p* = 0.001; Simpson: *F*_(3,__314)_ = 8.960, *p* = 0.003; Figure 3 and Supplementary Table 7). Though a significant driver of bacterial and fungal community structure, line could only explain between 0.8% (16S) and 1.1% (ITS) of variation in beta-diversity models (Supplementary Table 6). The explanatory power of line was well exceeded by that of plant ontogeny (early or late season); this factor explained 15% of fungal [tolerance model: *F*_(3,__314)_ = 59.359, *p* = 0.001] and 9% of bacterial [tolerance model: *F*_(1,__317)_ = 32.550, *p* = 0.001] variation in rhizosphere community composition. This effect was consistent across both tolerance and domestication models (Supplementary Table 6).

Progression from early to late season samples increased both fungal and bacterial alpha diversity for all metrics except fungal richness, which went unchanged over time [tolerance models: 16S richness: *F*_(1,__317)_ = 34.181, *p* < 0.001; ITS richness: *F*_(1,__314)_ = 0.067, *p* = 0.761; Supplementary Table 5]. Time of rhizosphere harvest also significantly impacted rhizosphere microbial biomass, as proxied by dsDNA concentrations [tolerance model: *F* = 64.56, *p* < 0.001; Supplementary Table 8]. Specifically, microbial biomass increased by 71.4% from early to late season time points (Supplementary Figure 5).

#### Tolerance categories and domestication contribute to subtle differences in overall rhizosphere community structure

**FIGURE 4:**
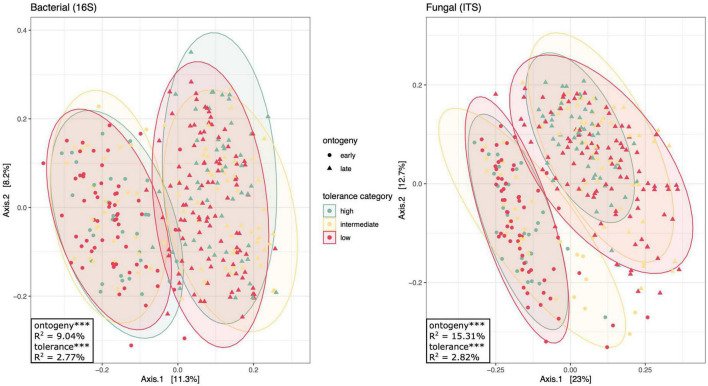
Principal coordinates analysis of bacterial and fungal communities separated by plant ontogeny (shape: early or late) and tolerance category (color: high, intermediate, or low). Drawn around each of the six ontogeny × tolerance category groups are 95% confidence ellipses. This figure uses the same ordination as Supplementary Figures 4, 6, but colors points by domestication status, rather than line (Supplementary Figure 4) or domestication (Supplementary Figure 6). Factors that explain a significant proportion of community variation as determined by a PERMANOVA of Bray–Curtis dissimilarity are displayed in the bottom-left corners (See Supplementary Table 6): *** denotes *p* < 0.001.

In order to compare rhizosphere communities associating with high and low tolerance plants, we assigned our four tomato lines to one of three tolerance categories (low, intermediate, or high; Figure 1). Perhaps unsurprisingly, since the tolerance categories consisted of between one and two lines, tolerance significantly influenced rhizosphere community composition [16S: *F*_(2,__317)_ = 4.986, *p* < 0.001; ITS: *F*_(2,__314)_ = 5.462, *p* < 0.001; Figure 4 and Supplementary Table 6]. Interestingly, tolerance explained slightly more variation in community composition models than tomato line alone (*R*^2^_*tolerance*_= 2.8% for both 16S and ITS). Tolerance also impacted species richness and evenness, with the exception of inverse Simpson diversity for bacterial communities [Shannon: *F*_(2,__317)_ = 2.960, *p* = 0.053; Richness: *F*_(2,__317)_ = 3.928, *p* = 0.021; Simpson: *F*_(2,__317)_ = 1.056, *p* = 0.349] and Shannon diversity for fungal communities [Shannon: *F*_(2,__314)_ = 2.222, *p* = 0.110; Richness: *F*_(2,__314)_ = 5.662, *p* = 0.004; Simpson: *F*_(2,__314)_ = 7.685, *p* = 0.001] (Supplementary Table 7). Our high tolerance line possessed 8.7% greater bacterial and 7.4% greater fungal species richness than low tolerance lines (Figure 3). Mean bacterial and fungal richness of intermediate tolerance rhizospheres fell between that of high and low tolerance lines.

In addition to comparisons across tolerance categories, we were interested in comparing rhizosphere community structure between wild and domesticated lines (Figure 1). Domestication was a significant predictor of community composition [16S: *F*_(2,__317)_ = 5.773, *p* < 0.001; ITS: *F*_(2,__314)_ = 6.529, *p* < 0.001; Supplementary Figure 6], but accounted for less variation than tomato line (16S: *R*^2^ = 1.6%; ITS: *R*^2^ = 1.1%; Supplementary Table 6). Like tolerance, domestication did not significantly affect Inverse Simpson diversity of bacterial communities, but did shape Shannon diversity and richness [Shannon: *F*_(1,__317)_ = 3.389, *p* = 0.067, Richness: *F*_(1,__317)_ = 7.166, *p* = 0.008; Simpson: *F*_(1,__317)_ = 0.487, *p* = 0.509]. In fungal communities, domestication exerted a significant or approaching-significant effect on all three alpha-diversity metrics [Shannon: *F*_(1,__314)_ = 3.483, *p* = 0.063; Richness: *F*_(1,__314)_ = 7.486, *p* = 0.007; Simpson: *F*_(1,__314)_ = 9.013, *p* = 0.003) (Supplementary Table 7). Wild lines harbored rhizosphere communities of 7.2% greater bacterial and 5.6% greater fungal richness than domesticated lines (Figure 3). The effect of domestication on biomass, as proxied by dsDNA concentrations, was also significant (*F* = 4.378, *p* = 0.037; Supplementary Table 8), with wild lines harboring rhizospheres of 12.0% greater biomass on average (Supplementary Figure 5).

#### Herbivory induces variable shifts in fungal and bacterial rhizosphere communities

Contrary to our expectations, we found no evidence for hornworm herbivory inducing shifts in rhizosphere bacterial community composition overall [*F*_(1,__317)_ = 0.830, *p* = 0.730] or in a time-point-specific manner [*F*_(2,__317)_ = 0.747, *p* = 0.889] (Supplementary Table 6). However, investigating a domestication × herbivory interaction [*F*_(1,__317)_ = 1.488, *p* = 0.041] revealed evidence for herbivory shifting bacterial community composition in domesticated lines [*F*_(1,__157)_ = 1.511, *p* = 0.037], but not wild lines [*F*_(1,__159)_ = 1.160, *p* = 0.230] (Supplementary Table 9). In terms of bacterial alpha-diversity, ‘Better Boy’ was the only line to experience significant herbivory-induced shifts, specifically in Shannon diversity (*F* = 7.262, *p* = 0.009 and richness (*F* = 8.481, *p* = 0.005), but not inverse Simpson diversity (Figure 3 and Supplementary Table 10). Specifically, herbivory depleted ‘Better Boy’ bacterial richness by 16.4% and Shannon diversity by 3.3%. The remaining three lines experienced no significant effect of herbivory on any of the three bacterial alpha-diversity metrics (Figure 3 and Supplementary Table 10).

Rhizosphere fungal community responses to herbivory mirrored many of the bacterial community responses. Fungal community composition did not respond to herbivory overall [tolerance model: *F*_(1,__314_ = 0.730, *p* = 0.742] or in a time point specific manner [tolerance model: *F*_(1,__314)_ = 0.531, *p* = 0.956] (Supplementary Table 6). While an interaction between line and herbivory existed in both tolerance and domestication models [tolerance model: *F*_(1,__314)_ = 1.8124, *p* = 0.044], it appeared to be driven by an effect of herbivory on *S. pimpinellifolium* community composition that approaches significance [*F*_(1,__77)_ = 1.864, *p* = 0.060; Supplementary Table 11]. Herbivory exerted no significant influence over community composition within the rhizospheres of the other three tomato lines. When considering alpha-diversity, herbivory once again shifted fungal community richness and Shannon diversity, but not inverse Simpson diversity, in a line-dependent manner. Herbivory depleted ‘Better Boy’ fungal richness, as it did with bacterial richness, by 15.7% (*F* = 14.515, *p* < 0.001). Herbivory also increased species richness in *S. pimpinellifolium* by 20.2% (*F* = 18.936, *p* < 0.001) and ‘Sioux’ by 8.5% (*F* = 4.580, *p* = 0.036). Fungal Shannon diversity increased by 6.2% in *S. pimpinellifolium* rhizospheres in response to herbivory (*F* = 7.888, *p* = 0.006). ‘Matt’s Wild Cherry,’ was the only line for which herbivory did not affect either of the three considered fungal alpha-diversity metrics (Figure 3 and Supplementary Table 10).

#### Low and intermediate tolerance rhizospheres share more differentially abundant taxa than intermediate and high tolerance rhizospheres

**FIGURE 5:**
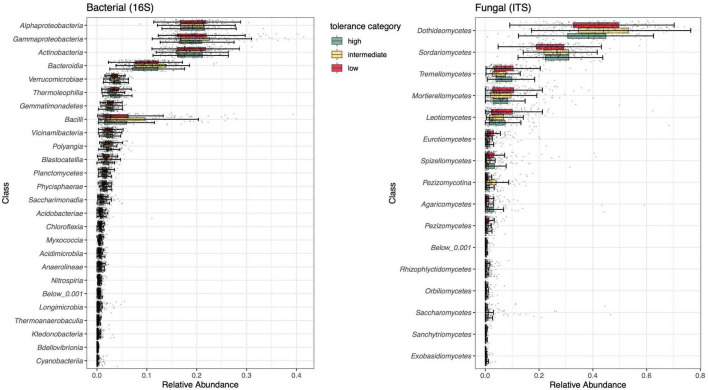
Summary of class-level rhizosphere composition of bacterial (left) and fungal (right) communities compared across tolerance categories (high, intermediate, low). Individual taxa (ASVs) were grouped by class, displayed on the *y*-axis. Low abundance classes (<0.001%) were grouped together in a single category. *n* = 38–40 for all treatments.

A broad assessment of microbial composition across herbivory treatments and time points indicated that bacterial communities were dominated by Gammaproteobacteria (19.9%), Alphaproteobacteria (19.3%), Actinobacteria (18.7%), and Bacteroidia (10.2%). All other bacterial classes occupied < 10% of relative abundance (Figure 5 and Supplementary Figure 7). The most abundant fungal classes were Dothideomycetes (50.0%) and Sordariomycetes (25.7%), with other classes occupying < 10% of relative abundance (Figure 5 and Supplementary Figure 7).

Following overall analysis of factors contributing to variation in rhizosphere community composition and diversity, we examined differentially abundant bacterial and fungal taxa between tolerance categories at the individual ASV level (Supplementary Tables 12, 13). Comparing the rhizosphere communities of the two low tolerance lines with those of the high tolerance line yielded the most differentially abundant taxa: 50 (33 bacterial and 17 fungal). Interestingly, despite belonging to opposite ends of the domestication spectrum used in this experiment, low and intermediate tolerance rhizospheres were distinguished by only 19 differentially abundant taxa (13 bacterial and 6 fungal). In contrast, intermediate and high tolerance lines, both of which are wild lines, were distinguished by 39 differentially abundant taxa (25 bacterial and 14 fungal).

Notable among the bacterial taxa differentially abundant between high and low tolerance lines were three members of *Sphingobacterium*, all of which were enriched in the rhizospheres of the high tolerance line (between 7- and 17-fold; Supplementary Table 12). Four members of Sphingomonadaceae, an unrelated family with relevance to tomato rhizospheres (Kaplan et al., 2020; Smulders et al., 2022), were also differentially abundant within these comparisons. Only one of these taxa, a *Sphingomonas* sp., was enriched in our high tolerance line (16-fold). All other differentially abundant Sphingomonadaceae were uniquely enriched in low and/or intermediate tolerance rhizospheres. These three comprise another *Sphingomonas* sp. (19-fold enriched in low and intermediate), one *Sphingopyxis* sp. (threefold enriched in low), and an *Elin6055* sp. (>21-fold enriched in intermediate compared to both low and high tolerance lines).

Within fungal communities, most differentially abundant taxa were enriched in either low or intermediate tolerance rhizospheres, but depleted in high tolerance rhizospheres (Supplementary Table 13). These include *Phallus rugulosus*, a saprobic stinkhorn fungus most enriched in low tolerance lines; *Alternaria iridiaustralis* and *Neosetophoma samararum*, two Pleosporales fungi that were similarly enriched (between 15- and 20-fold) in low and intermediate tolerance rhizospheres; *Cercospora sojina*, the fungal agent behind frogeye leaf spot in soybean, which was enriched in low and, to a lesser extent, intermediate tolerance lines; and *Filobasidium floriforme*, which was abundant in both low and intermediate tolerance rhizospheres. None of the differentially abundant bacterial or fungal ASVs are known tomato pathogens.

#### Herbivory shifts the abundance of fewer taxa in high tolerance rhizospheres than it does in low tolerance rhizospheres

We next identified individual ASVs that experienced significant shifts in relative abundance in the rhizosphere in response to herbivory (Supplementary Tables 14–17). Specifically, we used DeSeq2 to identify differentially abundant taxa between herbivory treatments within either tolerance categories or domestication. By performing these two suites of DeSeq2 analyses, we aimed to identify taxa that were both sensitive to herbivory and enriched in either high or low tolerance lines, and therefore a potential marker of either high tolerance or susceptibility.

Overall, hornworm herbivory affected the relative abundance of 14 microbial taxa (5 bacterial, 9 fungal) in the two low tolerance lines, 9 taxa in the intermediate tolerance line (7 bacterial, 2 fungal), and 7 taxa in the high tolerance line (6 bacterial, 1 fungal) (Supplementary Tables 14, 15). In the high tolerance line, hornworm herbivory notably depleted six of the seven differentially abundant fungal and bacterial ASVs (5/6 bacterial taxa, 1/1 fungal taxa). The one ASV that was enriched in response to herbivory in high tolerance rhizospheres belonged to *Sphingomonas* (21-fold enriched). In contrast, when considering herbivory-induced shifts in low tolerance rhizosphere communities, all but two taxa were enriched in response to herbivory (5/5 bacterial, 7/9 fungal). Among the enriched taxa was a strain of *N. samararum*, a Pleosporales fungus; this ASV was twofold enriched in response to herbivory in low tolerance lines, but 22-fold depleted in response to herbivory in the high tolerance line. Similarly, a bacterial *Pseudomonas* strain was sevenfold enriched in infested low tolerance rhizospheres, but 20-fold depleted in infested high tolerance rhizospheres (Supplementary Table 14).

When we identified differentially abundant taxa between hornworm herbivory treatments within wild and domesticated cohorts, domesticated lines experienced shifts in 14 ASVs (5 bacterial, 9 fungal), while wild lines experienced shifts in the relative abundance of only 5 ASVs (5 bacterial, 0 fungal) (Supplementary Tables 16, 17). Four of the ten instances of bacterial taxa responding to hornworm herbivory across wild and domesticated lines involve members of the Sphingobacteriaceae family. Previously, this family, particularly *Sphingobacterium* spp. was enriched in the high tolerance line (Supplementary Table 16). Here, one *Sphingobacterium* sp. was enriched in infested rhizospheres of both wild and domesticated lines, while another *Sphingobacterium* sp. was enriched only in wild infested rhizospheres. The other Sphingobacteriaceae member, a *Pedobacter* sp., decreased in abundance in infested wild rhizospheres.

## Discussion

Though several growth-promoting microbes have been implicated in plant expression of tolerance to herbivory (e.g., Barazani et al., 2007; Currie et al., 2011; Cosme et al., 2016; Frew et al., 2020), the characteristics of a whole rhizosphere microbiome that associates with tolerant plants are unknown. In this field experiment, wild tomato lines tolerated hornworm herbivory more successfully than domesticated cultivars, with ‘Matt’s Wild Cherry’ almost perfectly compensating for fitness losses to herbivory (Figure 1). Tomato rhizosphere communities were primarily shaped by the time point of rhizosphere harvest (i.e., plant ontogeny) and to a lesser extent by tomato line, tolerance categories, domestication, and occasionally herbivory. Overall, our results predict that rhizosphere community traits associated with high tolerance include: (1) higher species richness; (2) resistance to shifts in community composition, species richness, and evenness in response to herbivory; and (3) higher relative abundance of several ASVs within the *Stenotrophomonas*, *Sphingobacterium*, and *Sphingomonas* genera. When comparing patterns of differentially abundant taxa between uninfested and infested rhizospheres, we found that responses to herbivory within the high tolerance line were dominated by depletion of rhizosphere taxa (i.e., ASVs). In contrast, low tolerance rhizospheres more often showed enrichment of particular taxa as a consequence of herbivore infestation (Supplementary Tables 14–17). Taken together, these trends predict that excluding certain deleterious taxa from a rhizosphere may be just as, if not more, important as attracting beneficial taxa for modulating expression of tolerance to herbivory. They also contribute to the evidence accumulated in this study that a tolerance-associating tomato rhizosphere may be a stable rhizosphere, robust to stress-induced perturbation. Although the current study examined a small set of tomato lines, our results suggest key differences in the rhizosphere community that should be further explored to clarify the role root microbes play in in shaping tolerance.

The observed line-level variation in tolerance to tobacco hornworm defoliation fell largely within our prediction that wild lines would better tolerate herbivory compared to domesticated lines (Figure 1). In previous experiments, domesticated tomato cultivars have proven less tolerant to manual defoliation (Welter and Steggall, 1993) and generalist caterpillar herbivory (Paudel et al., 2019; Ferrero et al., 2020) than their wild relatives. Similarly, we saw domesticated lines suffer the largest (26%) yield loss in response to herbivory, followed by the wild *S. pimpinellifolium* (14% yield loss) and the domestication intermediate ‘Matt’s Wild Cherry’ (3% yield loss). Though fruit yield was often sensitive to herbivory treatment, hornworm herbivory had a less consistent impact on fruit weight. Significant changes in fruit weight were seen only in *S. pimpinellifolium* plants of the truncated lifespan group, which captured 4 weeks of fruit production beginning at the onset of fruiting (Figure 1). Though significant, this difference only amounted to a 5% increase in fruit weight within infested plants. In other work, manual defoliation has significantly reduced fruit weight in tomato cultivars as well as the domestication intermediate ‘Matt’s Wild Cherry’ (Welter and Steggall, 1993). The absence of such a response in this experiment may be explained by less severe defoliation (50 vs. 70%) or enhanced plant compensatory capacity to hornworm damage compared to mechanical damage (Korpita et al., 2014). Contrary to previous work in solanaceous plants (Welter and Steggall, 1993; Schwachtje et al., 2006) we also found no evidence for any tomato lines extending their fruiting window to cope with herbivory (Figure 2 and Supplementary Figure 1).

Our primary objective in this study was to identify bacterial and fungal rhizosphere community characteristics associated with tolerance to herbivory in our four considered tomatoes. Initially, we predicted that high tolerance lines would harbor high diversity rhizospheres. In line with our expectations, our high tolerance ‘Matt’s Wild Cherry’ possessed the highest fungal and bacterial species richness (Figure 3). This line harbored rhizospheres of 8.7% greater bacterial richness and 7.4% greater fungal richness than low tolerance cultivars, predicting that overall higher species richness may be a signature of a tolerant rhizosphere. On broader scales, there is support for a positive relationship between soil health and microbial diversity (Nielsen et al., 2015; Delgado-Baquerizo et al., 2016), though network analyses may offer more nuance (Wei et al., 2015; Gómez-Aparicio et al., 2022). By fostering high competition and niche overlap, microbial diversity is thought to buffer against the invasion of pathogens (van Elsas et al., 2012; Wei et al., 2015), which could indirectly support plant tolerance by safeguarding plants against a defense response that would divert resources away from reproduction and/or compensation. Greater microbial diversity may also translate to more extensive functional capabilities that could contribute to the expression of tolerance, although some research indicates there are limitations to the benefits of increased diversity within microbiomes (French et al., 2021b).

Our results further showed that the tomato line expressing high tolerance to herbivory also harbored a more stable root microbial community, which is the opposite of our initial prediction that high-tolerance-associating rhizospheres would exhibit greater herbivore-induced changes. Instead, hornworm herbivory did not affect rhizosphere bacterial and fungal community structure in wild lines, which expressed either intermediate or high tolerance. Herbivory did, however, induce significant changes in bacterial community composition in the rhizospheres of the two low tolerance domesticated cultivars (Supplementary Table 9). Microbial species richness and evenness was also unaffected by herbivory in the high tolerance ‘Matt’s Wild Cherry’ (Figure 3). In contrast, the three other lines exhibited either depletion of rhizosphere species richness, in the case of ‘Better Boy,’ or species enrichment, in the cases of *S. pimpinellifolium* and ‘Sioux,’ in response to herbivory (Figure 3). Similar to these findings, the magnitude of pathogen-induced changes to bacterial and fungal rhizosphere communities was greater in cultivated rice than in their wild relatives, which were also more resistant to pathogen invasion (Shi et al., 2018). Rhizosphere community stability in the face of biotic stresses such as herbivory could therefore represent an important feature of tolerance-associating microbiomes and stress-tolerant rhizospheres in general. However, *S. pimpinellifolium* bacterial rhizosphere composition has been shown to shift with aphid feeding (French et al., 2021a), suggesting the effects of herbivory stress may depend on feeding guild or species (Malacrinò et al., 2021). Our results predict that, in the case of tolerance, stress-induced microbiome recruitment predicted under the “cry for help” hypothesis (Rolfe et al., 2019) could involve subtle but functionally impactful changes rather than large-scale restructuring of rhizosphere composition. Further characterization of rhizosphere responses to herbivory using additional wild and domesticated genotypes and herbivore species is needed to clarify how community stability may contribute to tolerant responses.

In addition to community-level rhizosphere traits, we identified microbial taxa that were both herbivory-responsive and significantly richer in the rhizospheres of high tolerance plants (Supplementary Tables 12–17). By identifying genera that are both (1) more abundant in the high tolerance line and (2) enriched in response to hornworm infestation, we sought to acquire a shortlist for microbial taxa that may be involved in responding to herbivory and supporting tolerance. Three bacterial genera possessed such herbivory-sensitive, high-tolerance-associating ASVs: *Stenotrophomonas*, *Sphingobacterium*, and *Sphingomonas*. Briefly, *Stenotrophomonas* spp. have proven sensitive to herbivory in other systems, specifically *Brassica* spp. facing whitefly (Kong et al., 2016) and cabbage root fly herbivory (Ourry et al., 2018). Many members in this genus have plant-growth-promoting interactions with tomato, notably involving reshaping fungal networks (Schmidt et al., 2012), promoting growth (Schmidt et al., 2012; Alijani et al., 2020; Manh Tuong et al., 2022), protecting against pathogens (Marina et al., 2019; Alijani et al., 2020), and conferring resistance to generalist herbivory (Ling et al., 2022). *Sphingomonas* spp., and generally Sphingomonadaceae, appear to be signatures of tomato rhizospheres across tomato domestication (Kaplan et al., 2020; Smulders et al., 2022). Members of this genus, too, can promote plant growth in wild (Khan et al., 2017) and domesticated (Halo et al., 2015) tomatoes, particularly under salinity stress. In our experiment, *Sphingobacterium* spp. also frequently appeared as unique responders to hornworm herbivory and signatures of high tolerance rhizospheres. Specifically, three *Sphingobacterium* spp. were enriched in high tolerance rhizospheres, and four of the ten instances of bacterial taxa responding to hornworm herbivory across wild and domesticated lines involved members of Sphingobacteriaceae. Members of the *Sphingobacterium* genus have been involved in biofilm formation that promotes tomato growth (Kalam et al., 2017) and plant oxidative stress mitigation in response to abiotic stressors (Vaishnav et al., 2020). Whether *Sphingobacterium* spp., or members of other identified genera, could confer similar benefits to host plants under biotic stresses such as herbivory deserves additional attention. Beyond their individual contributions to plant growth promotion, it is possible that some of these taxa also created stability and connectivity in the rhizosphere, and network analyses considering these taxa in tomato rhizospheres may offer a more detailed understanding of their contributions to a tolerant rhizosphere. Interestingly, no fungal genera contained these herbivory-sensitive, high-tolerance-associating ASVs. Instead, several fungal ASVs were herbivory-sensitive and low-tolerance-associating, suggesting a role in susceptibility to herbivory.

## Conclusion

In this investigation of whole rhizosphere community traits associating with herbivore tolerance in tomatoes, we found evidence for tolerance-associated rhizospheres possessing high species richness, community stability during herbivory, and an abundance of certain *Stenotrophomonas* spp., *Sphingomonas* spp., *Sphingobacterium* spp. Collectively, these results suggest that tolerance-associated root microbial communities may be more robust to perturbation during stressful events like herbivory, and may be assembled using mechanisms that both recruit beneficials and exclude harmful taxa. Additional work that considers a wider variety of wild and domesticated (and/or high and low tolerance) lines is necessary to evaluate whether the rhizosphere community traits associated with our tolerant line here extend to other tolerant lines. In addition, further work examining the attributes of tolerance-associated microbiomes in other systems would help clarify whether the results from the current study are specific to tomatoes or also found in other crop plants expressing tolerance to herbivory. Such research would support efforts to develop cultivars and microbial amendments that represent sustainable strategies for managing pests in agroecosystems.

## Data availability statement

The datasets presented in this study can be found in online repositories. The names of the repository/repositories and accession number(s) can be found below: https://www.ncbi.nlm.nih.gov/PRJNA849200.

## Author contributions

ET, IK, and LE conceived and designed the study and wrote and revised all drafts of the manuscript. ET conducted the field study, collected all data, performed all statistical analyses, and developed all figures. All authors contributed to manuscript revisions and approved the final manuscript.
