# Supplementary material for: Characterizing rhizosphere microbial communities associated with tolerance to aboveground herbivory in wild and domesticated tomatoes
Source: Front Microbiol. 2022 Sep 14;13:981987. doi: 10.3389/fmicb.2022.981987 (PMC9515613; doi:10.3389/fmicb.2022.981987)
Supplement: Supplementary file 2 [file Data_Sheet_1.DOCX]

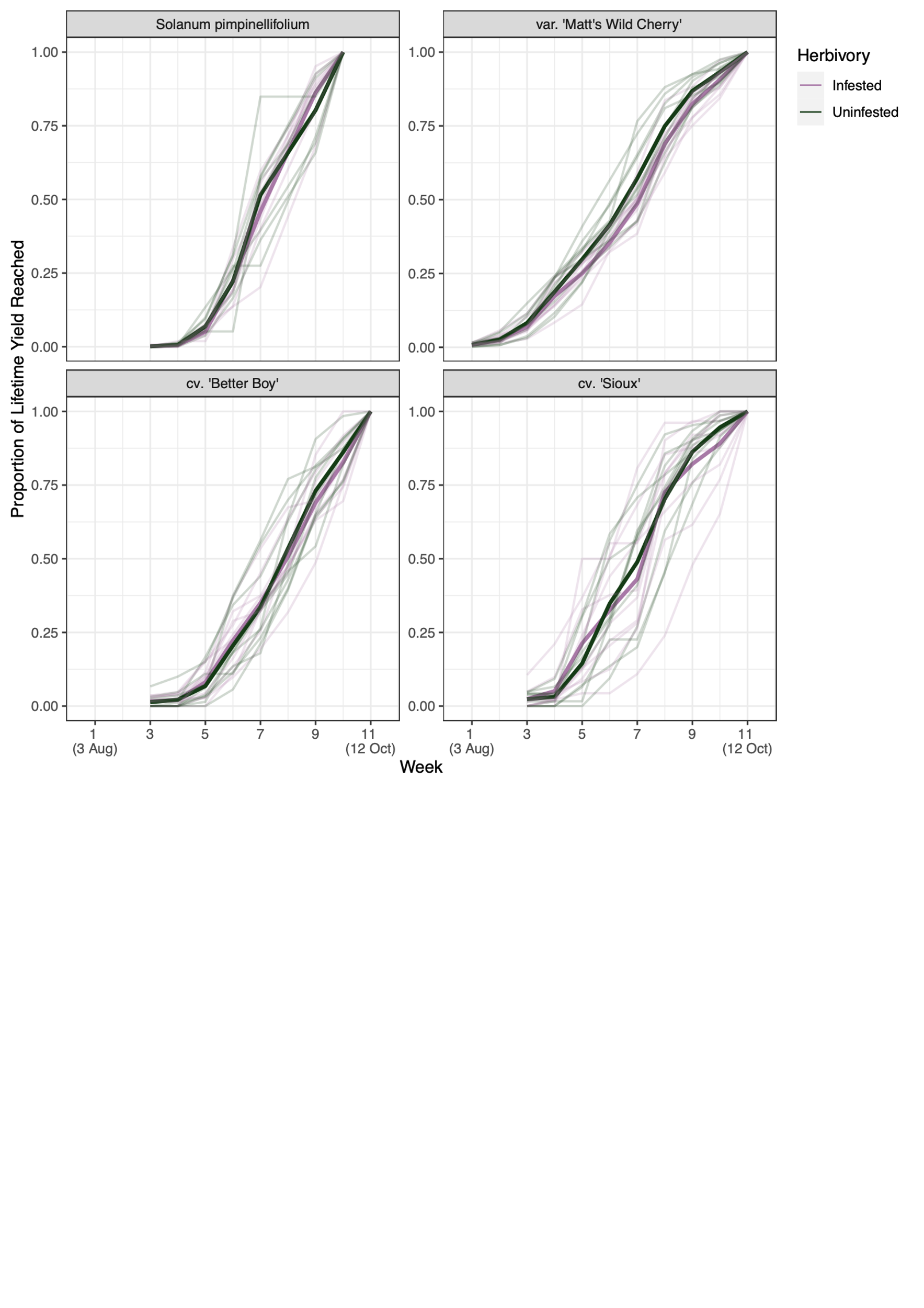


**Figure S1**. Accumulation curves reflecting trends in lifetime fruit output among the four tomato lines. Thicker lines represent mean cumulative sums of lifetime yield for the two herbivory treatments (infested or uninfested). Fainter lines track the proportion of a lifetime yield expressed in a given week for a single plant, with all plants of the *n* = 10 full lifetime blocks represented. We predicted that uninfested (green) lines would plateau in the late season while infested (purple) lines continued to increase, reflecting extension of a fruiting window. However, no such shifts in a fruiting window were observed.

**Figure S2**. Heatmaps of calcium, cation exchange efficiency (CEC), magnesium, organic matter, soil pH, phosphorus, and potassium estimates from bulk soil samples collected during rhizosphere harvests. Each heatmap represents the field layout, with cardinal directions on the x and y axes, and each tile representing one experimental block. Blank white tiles represent the *n* = 10 full lifetime blocks that were never removed for rhizosphere sampling, and therefore no bulk soil was collected from these blocks.


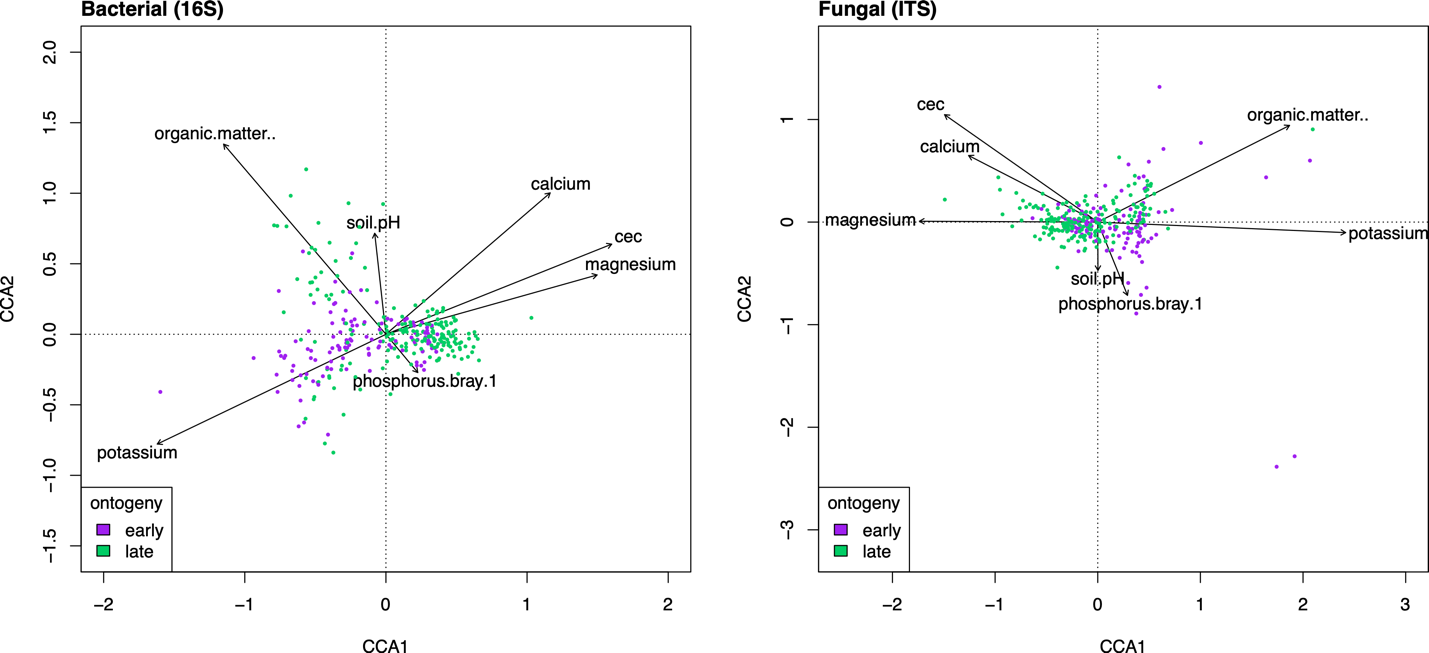


**Figure S3**. Canonical correspondence analysis of the extent to which soil nutritional factors (calcium, cation exchange efficiency [CEC], magnesium, organic matter, soil pH, phosphorus, and potassium; constraining variables) explain variation in bacterial and fungal community Bray–Curtis dissimilarity (response variable). Point color reflects plant ontogeny, or the timepoint at which the plant’s rhizosphere was destructively sampled (early or late). See Tables S4 and S5 for relevant statistical summaries.


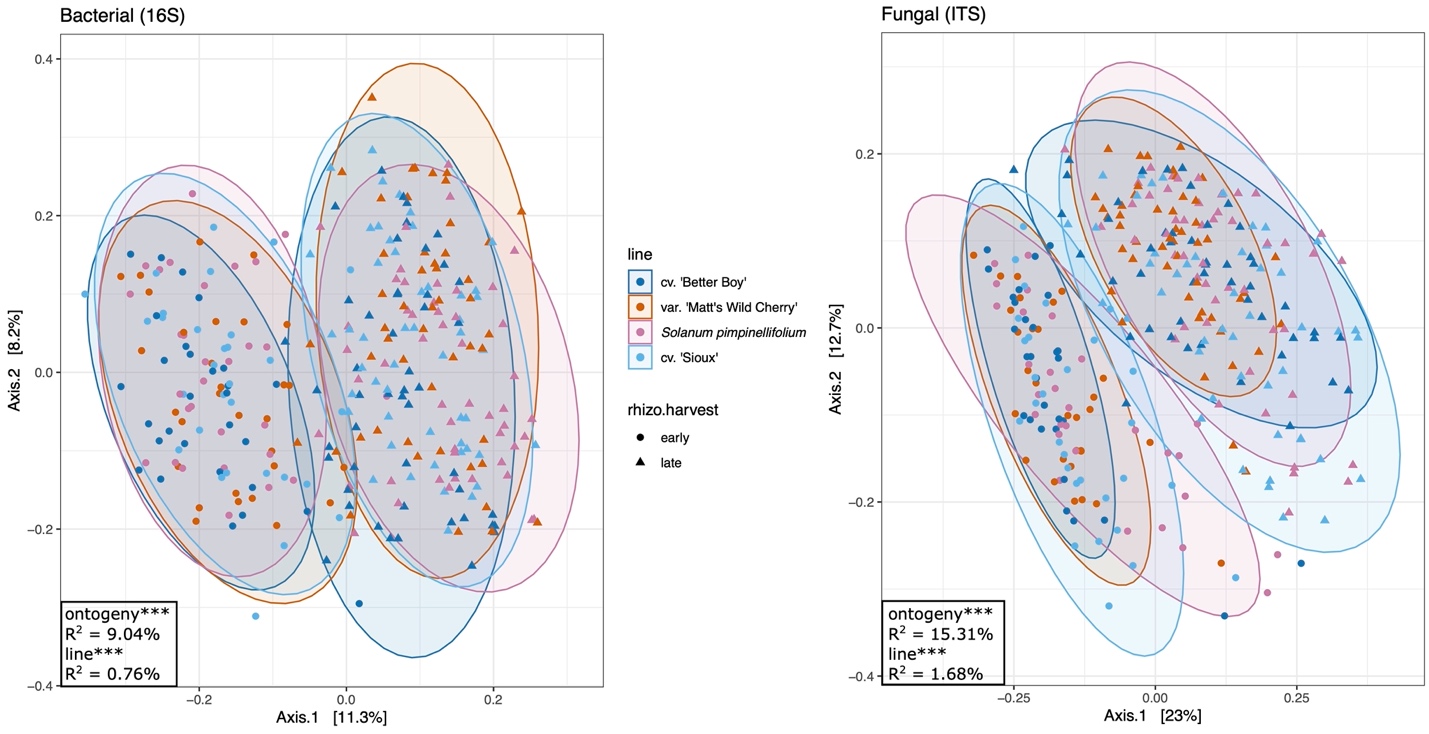


**SP**

**SP**

**Figure S4**. Principle coordinates analysis of bacterial and fungal communities separated by plant ontogeny (shape) and line (color). Drawn around each of the six ontogeny × tomato line groups are 95% confidence ellipses. This figure uses the same ordination as Fig. 3 and S6, but colors points by line, rather than tolerance category (Fig. 3) or domestication (Fig. S6). Factors that explain a significant proportion of community variation as determined by a PERMANOVA of Bray–Curtis dissimilarity are displayed in the bottom-left corners (see Table S6): *** denotes *p* < .001. Reported statistics reflect models nesting line within tolerance.


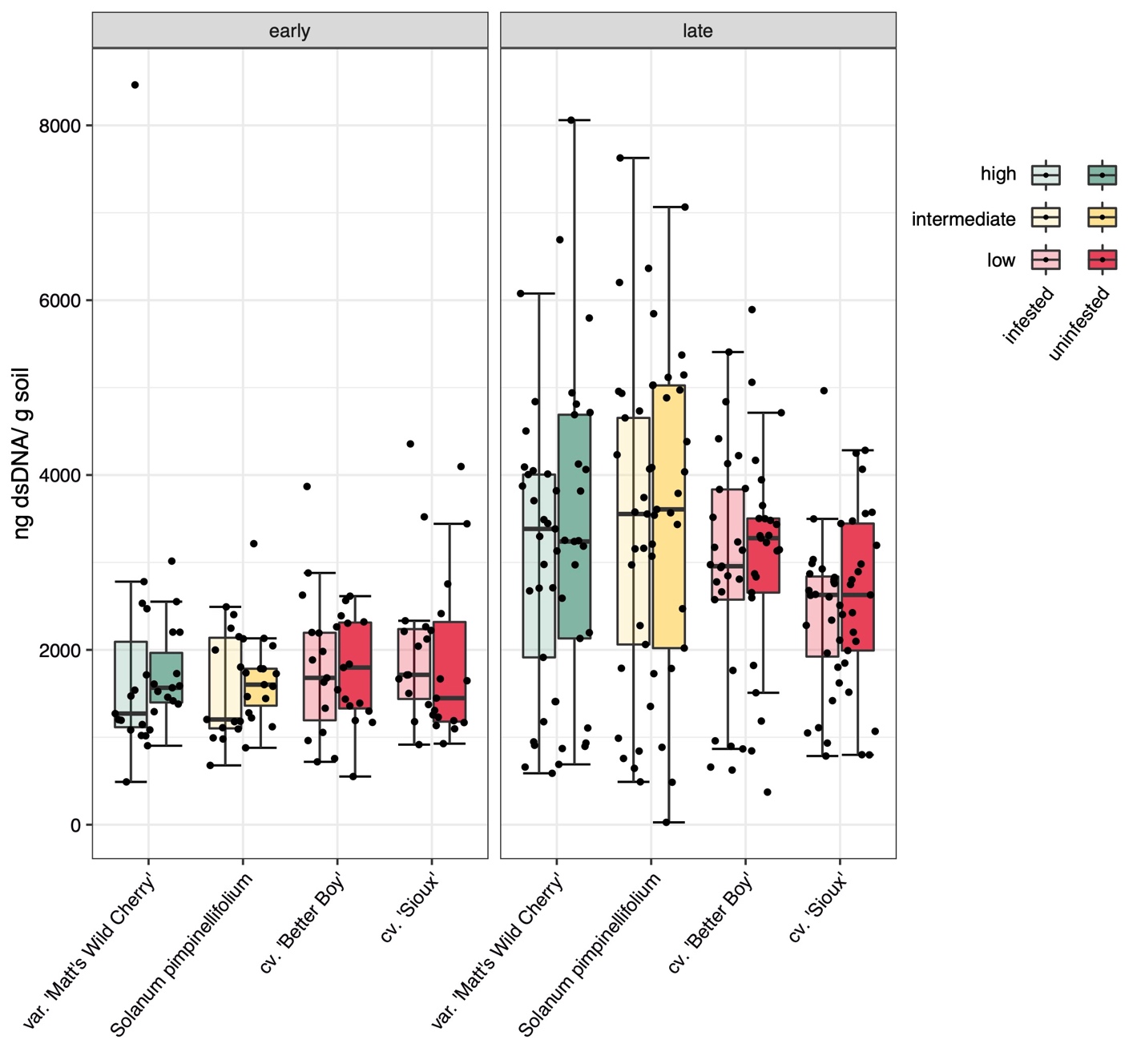


**Figure S5**. Comparison of overall microbial biomass of rhizosphere communities collected from infested and uninfested plants across plant ontogeny (early or late). Microbial biomass was estimated using PicoGreen dsDNA concentration (ng/μl) and adjusted by standardizing ng/μl concentrations to weight of soil samples used for extraction. Color reflects tolerance categories (high: green; intermediate: yellow; low: red) determined using yield data (Fig. 1) and shade reflects herbivory treatment (infested or uninfested). See Table S8 for relevant statistical summaries.


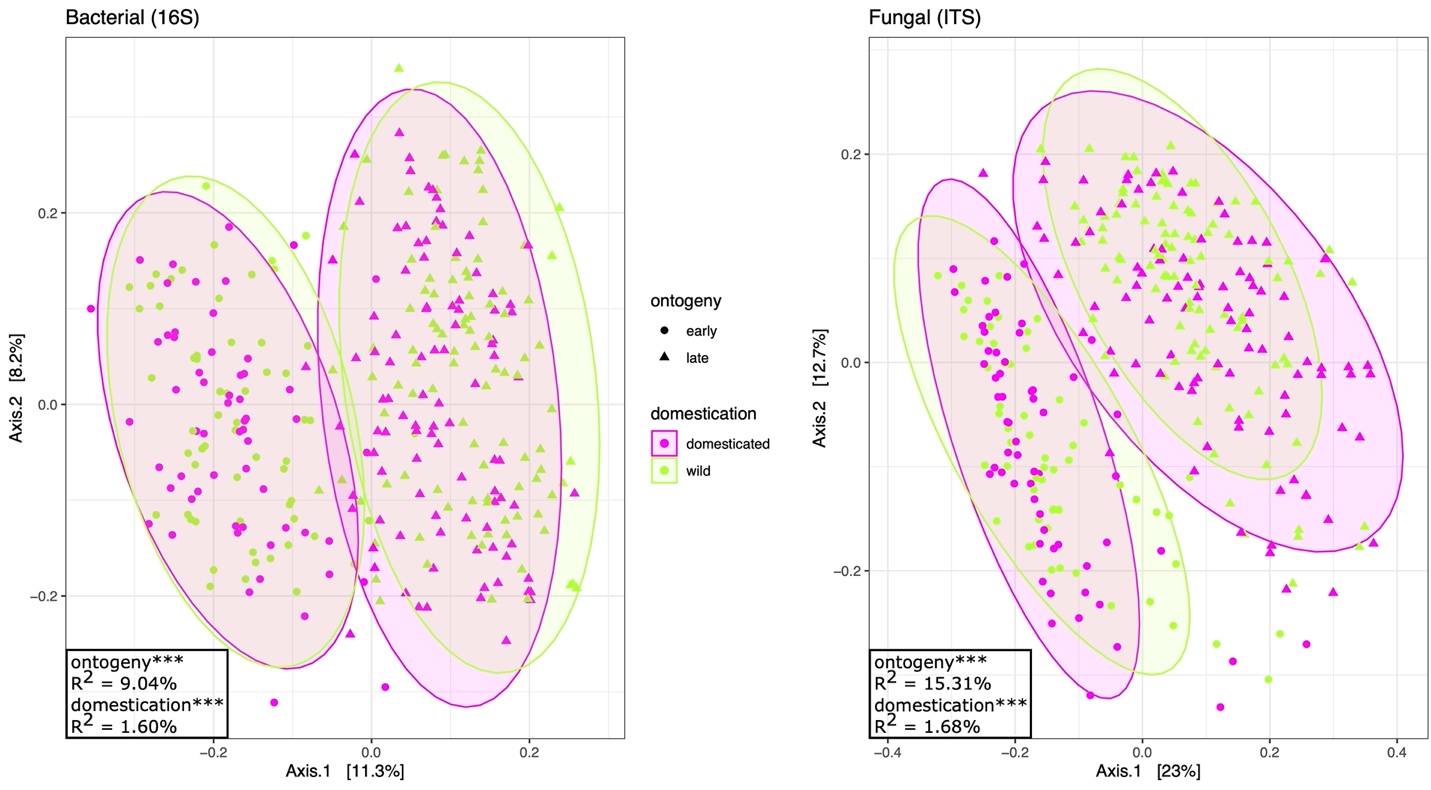


**Figure S6**. Principle coordinates analysis of bacterial and fungal communities separated by plant ontogeny (shape: early or late) and domestication status (color: wild or domesticated). Drawn around each of the six ontogeny × domestication groups are 95% confidence ellipses. This figure uses the same ordination as Fig. 3 and S4, but colors points by domestication status, rather than line (Fig. S4) or tolerance category (Fig. 3). Factors that explain a significant proportion of community variation as determined by a PERMANOVA of Bray–Curtis dissimilarity are displayed in the bottom-left corners (see Table S6): *** denotes *p* < .001.


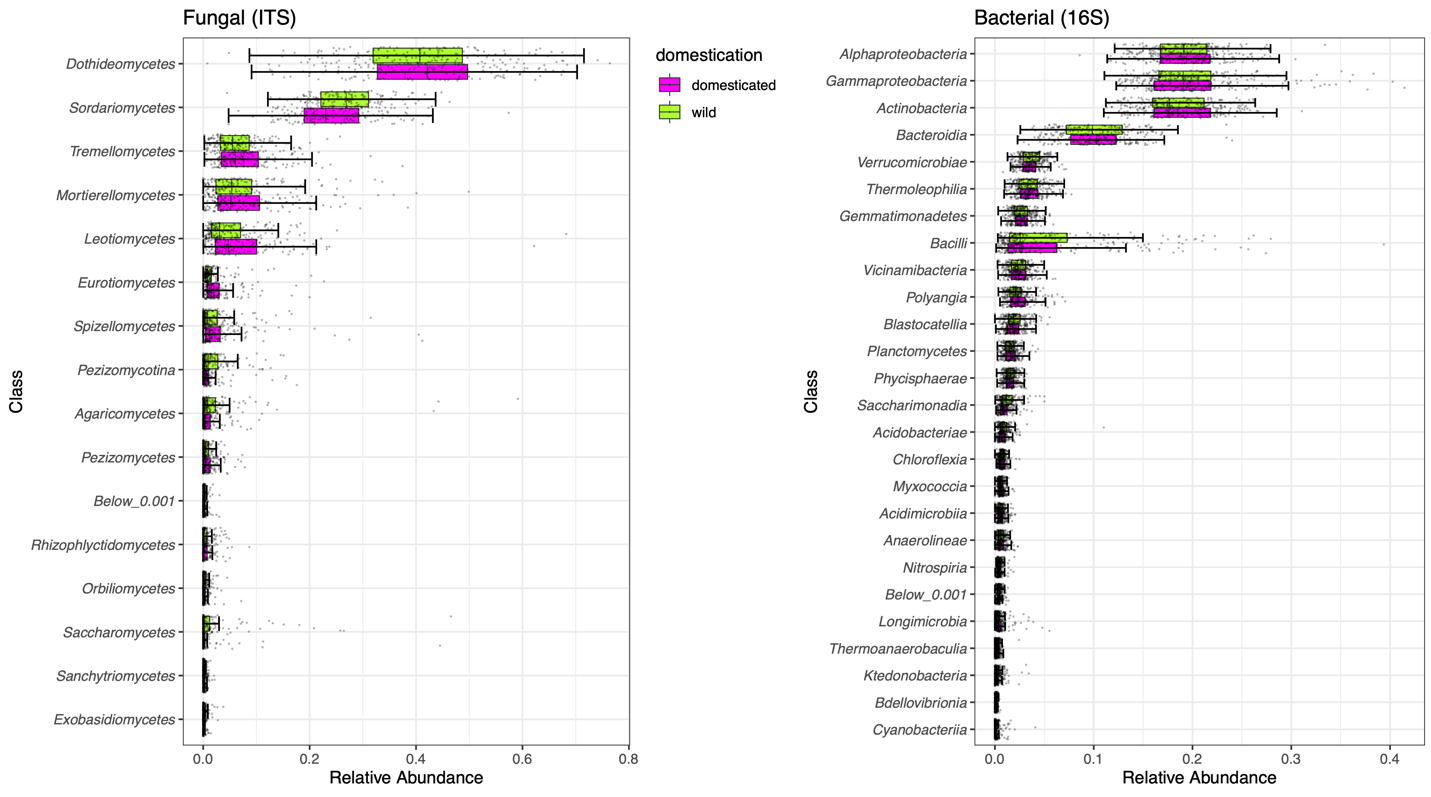


**Figure S7**. Class-level summary of rhizosphere composition of bacterial and fungal communities across domestication status, distinguished by bubble color. Individual taxa (ASVs) were grouped by class, displayed on the y-axis. Low abundance classes (< 0.001%) were pooled together, indicated by the “Below_0.001” category. n = 38–40 for all treatments.
